# Three-spined sticklebacks do not respond behaviorally to environmentally relevant concentrations of the toxic explosive 2,4,6-trinitrotoluene

**DOI:** 10.1007/s10646-026-03169-7

**Published:** 2026-10-23

**Authors:** Joy Willmer, Hendrik Schubert, Maj-Britt Hölzel, Niklas Stegmann, Ulrike K. R. Kammann, Jörn Peter Scharsack

**Affiliations:** 1 Thünen Institute of Fisheries Ecology, Herwigstraße 31, 27572 Bremerhaven, Germany; 2 University of Bremen, Bibliothekstraße 1, 28359 Bremen, Germany

**Keywords:** Marine dumped munitions, explosives, trinitrotoluene, TNT, avoidance behavior, *Gasterosteus aculeatus*

## Abstract

Approximately 1.6 million tons of conventional munitions were dumped in German coastal waters. Corrosion of munition shells facilitates the leakage of explosives, such as the toxic and carcinogenic 2,4,6-trinitrotoluene (TNT), into the marine environment. Consequently, TNT contamination was detected in seawater, sediments, and marine organisms near munition dump sites, and is known to cause adverse effects in marine organisms. Accordingly, avoidance of contaminated areas would be advantageous for fish. We therefore hypothesized that fish respond to dissolved TNT and exhibit an avoidance reaction. To test this, we exposed three-spined sticklebacks (*Gasterosteus aculeatus*) to environmentally relevant TNT concentrations and evaluated their behavioral response. Individual fish were placed in a behavioral test tank equipped with two hideout zones composed of artificial plants. The hideout zone occupied by the stickleback was infused with either TNT solution (100 µg/L) or control water. Behavioral readouts included the time stickleback spent in the infused hideout zone, time until the exposed hideout zone was first left, and number of transitions between hideout zones. Sticklebacks did not exhibit significant behavioral responses to environmentally relevant TNT exposure. Consequently, fish inhabiting munition dump sites may experience chronic TNT exposure, potentially increasing long-term toxicological risks.

**Supplementary Information:**

The online version contains supplementary material available at https://doi.org/10.1007/s10646-026-03169-7.

## Introduction

After the Second World War, approximately 1.6 million tons of conventional munitions were dumped in German coastal waters as part of efforts to demilitarize Germany (Böttcher et al. 2011). Conventional munitions refer to military weapons that contain explosives or incendiary substances and exert their effects through detonation or ignition, such as bombs, grenades, and mines (Bełdowski et al. 2020). Of this 1.6 million tons of conventional munitions, around 1.3 million tons are estimated to lay on the seabed of the North Sea and 300,000 tons in the Baltic Sea (Böttcher et al. 2011). While at least 15 official dump sites have been documented, the precise locations of much of the dumped munitions in North Sea and Baltic Sea remain uncertain due to imprecise navigation records, displacement by currents or fishing activity, and cases where munitions were already discarded on the route to the designated dump sites (Benn et al. 2010; Nakayama 2008).

To date, the metal shells of the munitions are exposed to salt water and corrosion for more than 70 years, resulting in the leakage of explosives into the marine environment (Beck et al. 2018, 2025; Voie and Mariussen 2017). One of the main explosives detected close to dumped munitions is 2,4,6-trinitrotoluene (TNT), which is among the most prevalent explosives used in conventional munitions (Böttcher et al. 2011). TNT is a nitroaromatic compound that degrades into the metabolites 2-amino 4,6-dinitrotoluene (2-ADNT) and 4-amino-2,6-dinitrotoluene (4-ADNT) (Koske et al. 2020). TNT concentrations measured at munition dump sites depend on the distance to the munitions (Beck et al. 2019). Concentrations of up to 3100 µg/L TNT were measured in close proximity to corroding munitions (< 1 cm) where gun wool material (explosives matrix) was freely accessible. However, concentrations decline rapidly with increasing distance from the munitions, with 16 µg/L at 1 cm distance to 3.3 µg/L at 50 cm distance (Beck et al. 2019).

Studies have confirmed the uptake of TNT by flatfish collected near munition dump sites, including common dab *Limanda limanda*, European flounders *Platichthys flesus*, and European plaice *Pleuronectes platessa* (Kammann et al. 2024, 2025; Koske et al. 2020). TNT and its metabolites were found in different biological matrices, such as urine, bile (Kammann et al. 2025), and muscle tissue (Maser et al. 2023, 2024). Moreover, TNT was detected in sessile organisms, such as seaweed, mussels, and sea stars (Beck et al. 2022).

The uptake of TNT is of critical concern, as studies have demonstrated its toxicity to a wide range of marine organisms including fish, like juvenile sheepshead minnows (*Cyprinodon variegatus*) and fathead minnows (*Pimephales promelas*) (Lotufo et al. 2010), crustaceans such as opossum shrimp (*Americamysis bahia*) (Ek et al. 2008), bivalves like blue mussels (*Mytilus edulis*) (Schuster et al. 2021), Atlantic purple sea urchin (*Arbacia punctulata*) and green macroalga (*Ulva fasciata*) (Nipper et al. 2001). For fish, the median lethal dose of TNT (LD_50_), at which 50% of the population dies after a specific test duration, varies depending on species and experimental conditions (Bailey et al. 1985; Bailey and Spanggord 1983; Talmage et al. 1999). For instance, fathead minnows were exposed to a range of TNT concentrations (100 − 10 000 µg/L) (Yoo et al. 2006). The calculated LD_50_ were 2700 µg/L for 96 h and 2200 µg/L for 240 h exposure and TNT exposure significantly reduced survival in both experimental trials. However, minnows exposed to concentrations above 3500 µg/L over 240 h developed spinal deformities and showed a reduced body mass and mortality (Yoo et al. 2006). Rainbow trout (*Onchorhynchus mykiss*) exhibited a LD_50_-96 h at 800 µg/L (Liu et al. 1983), whereas LD_50_-96 h values for bluegill sunfish (*Lepomis macrochirus*) ranged from 2000 µg/L (Nay et al. 1974) to 2800 µg/L depending on temperature conditions (Pederson 1970).

At munition dump sites, TNT concentrations in the acutely toxic range for fish (> 1000 µg/L) were only detected in close proximity (< 1 cm) to open explosive matrix (Beck et al. 2019). However, also TNT concentrations at and below 1000 µg/L cause negative effects on fish health (e.g.: Koske et al. 2019; Mariussen et al. 2018). In Atlantic salmon (*Salmo salar*), exposure to 1000 µg/L TNT for 48 h caused significant effects on blood physiology parameters and internal alterations in the dorsal muscle tissue (Mariussen et al. 2018). In zebrafish (*Danio rerio*) embryos, lethal and sublethal effects of TNT were observed after 120 h of exposure to 500–10 000 µg/L, with a concentration-dependent increase in chorda deformities (Koske et al. 2019). At concentrations as low 100 µg/L, TNT induced significant DNA damage in zebrafish embryos during 48 h of exposure (Koske et al. 2019). The latter TNT concentration lies within the concentrations measured at munition dump sites in the decimeter-scale vicinity of exposed explosive material (Beck et al. 2019). Therefore, the ability to detect and avoid TNT at such environmentally relevant concentrations could be advantageous for fish.

However, studies on behavioral responses of fish to TNT are scarce. To date, only a study by Smock et al. (1976) reported concentration-dependent behavioral alterations in fathead minnows exposed to TNT concentrations ranging from 1780 to 9540 µg/L (Smock et al. 1976). The minnows displayed a range of behavioral pattern, such as shock responses, skittering behavior, loss of equilibrium, and eventually mortality at concentrations above 3840 µg/L, presumably a response to acute intoxication (Smock et al. 1976). The present study did not intend to trigger acute toxicity in the experimental fish and therefore avoided high TNT concentration as tested by Smock et al. (1976).

One of the most frequently observed behavioral response of fish to pollutants in water is avoidance of contaminated areas (Araújo et al. 2016; Hansen et al. 1999; Hartwell et al. 1987, 1989; Robinson 2009; Silva et al. 2018; Sloman and McNeil 2012; Sprague 1968; Sprague and Drury 1969; Svecevičius 1999; Tierney 2016). For instance, Hartwell et al. (1989) investigated whether golden shiners (*Notemigonus crysoleucas*) avoid harmful metal concentrations before reaching lethal levels. Results showed that avoidance behavior occurred upon exposure with chromium, copper and arsenic at concentrations below the LD_50_ (Hartwell et al. 1989).

To the best of our knowledge, no studies have yet investigated whether fish display an avoidance behavior to TNT in water. The aim of the present study was to simulate a realistic TNT concentration near dumped munitions and to test whether fish exhibit behavioral responses to TNT before exposure reaches acutely toxic levels. Based on TNT concentrations measured at munition dump sites (Beck et al. 2019), an starting concentration of 100 µg/L was chosen for the present study, which was diluted once entering the experimental tank. This concentration falls within the range of environmentally relevant TNT concentrations recorded at dump sites and represents an exposure scenario that fish may encounter near dumped munitions. We therefore conducted behavioral tests with three-spined sticklebacks (*Gasterosteus aculeatus*) as model species.

Three-spined sticklebacks are common across the Northern Hemisphere, including North and Baltic Sea (Olin et al. 2022; Olsson et al. 2019; Schaarschmidt and Jürss 2003) and are a frequently used model species, as they are a physically and behaviorally resilient species (Huntingford and Ruiz-Gomez 2009). Based on the potential negative effects of TNT exposure, we hypothesized that fish behaviorally respond to TNT and might avoid exposure. To test this hypothesis, custom-build aquaria were used with hideout zones at either side enabling the three-spined stickleback to choose between hideouts with and without TNT contamination (infusion of 100 µg/L TNT). If the three-spined sticklebacks responded to TNT and avoided it, we expected them to move from the infused (increasingly contaminated) hideout to the uncontaminated hideout on the opposite side of the tank and remain there.

## Materials and methods

### Experimental fish

Experimental three-spined sticklebacks originated from parental sticklebacks collected in April 2023 from the Weser estuary at the Luneplate in Bremerhaven, Germany (53°28’36.9” N; 8°31’08.9” E). To the best of our knowledge, no intentional munition dumping has occurred at this site. Nevertheless, given its proximity to Bremerhaven, which was heavily bombed during World War II (Ernst 2014), the potential release of TNT from unexploded ordnance cannot be ruled out. Fertilized eggs were obtained by in-*vitro* fertilization (Barber and Arnott 2000). After hatching, larvae were first fed daily with *Artemia salina* nauplii and six months post-hatching with frozen mosquito larvae (*Chironomidae*) three times a week. The three-spined sticklebacks were housed in gender-mixed groups of approximately 30 individuals in 60 L tanks connected to a recirculation system equipped with a bio-filtration unit at 16 °C and 12/12 h light/ dark cycles. Two independent experiments were conducted, each comprising 60 experimental three-spined sticklebacks (*n* = 30 Control; *n* = 30 TNT exposure). The first experiment 1 was performed in August 2024, and the second experimental 2 in February 2025, using identical experimental conditions. All experimental fish were laboratory-hatched in April 2023. Consequently, fish were 16 months old during experiment 1 and 22 months old during experiment 2.

Experimental animals were treated under EU and German legislation according to animal testing application requirements with ethical approval by the local animal welfare authorities, project number 176 500-427-103-1/2020-2-5.

### Experimental setup

**Fig. 1:**
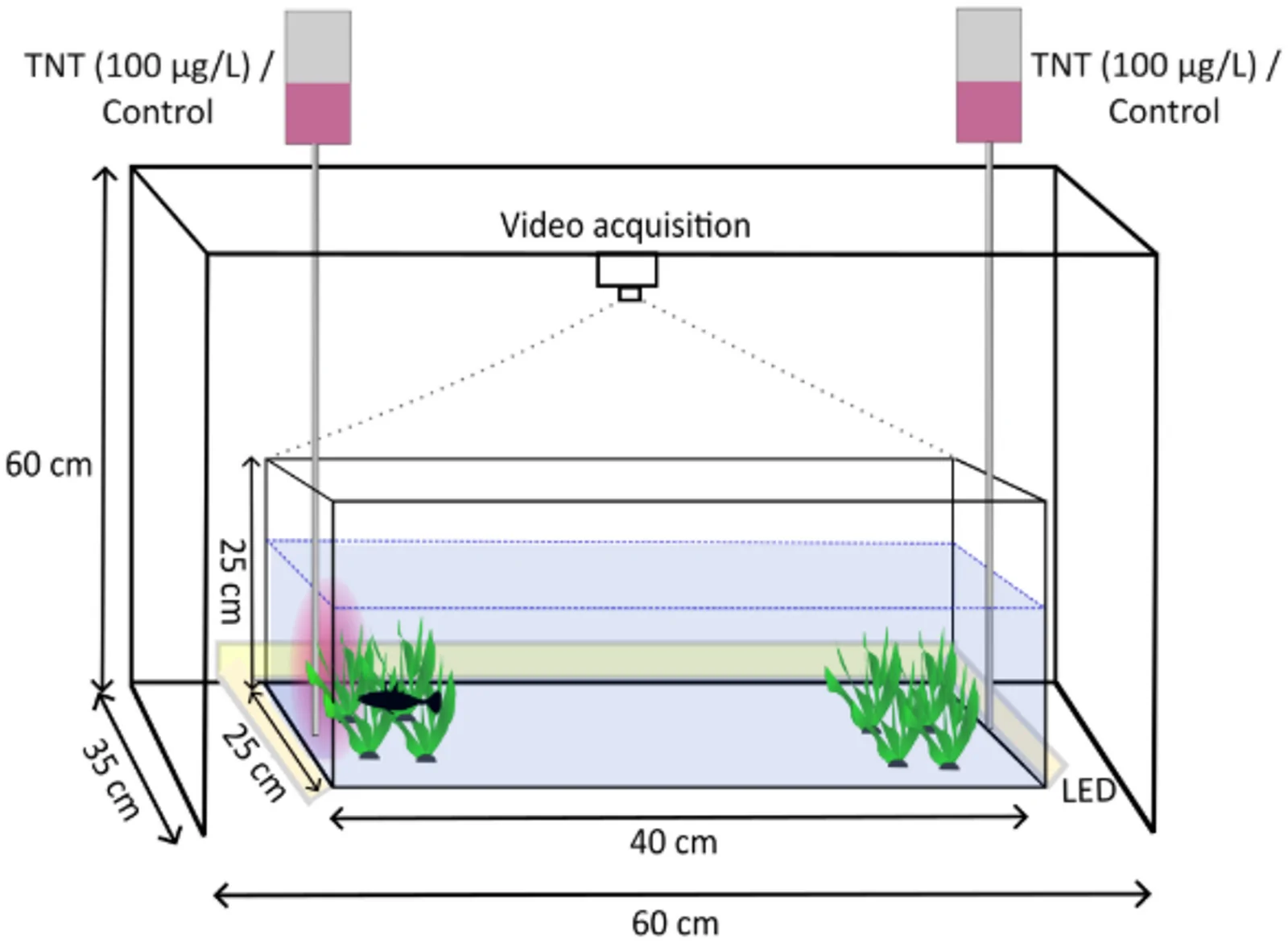
Experimental setup to assess the behavioral response of three-spined sticklebacks to TNT exposure: a glass tank containing two hideouts each composed of four artificial plants was filled with 10 L of holding tank water. Identical infusion systems were installed on opposite ends of the tank. When a three-spined stickleback had chosen a hideout, either TNT solution or the control solution was infused to the respective hideout. The system was equipped with a webcam for video recording and a black cloth cover to cover the setup during observations

Two identically equipped glass tanks (40 cm × 25 cm × 25 cm) were used for the avoidance assay, with one tank assigned to the TNT treatment and the other one to the control to avoid cross-contamination. To simulate hideout areas, four identical artificial plants were mounted on either side of the tank (Fig. 1). The inner walls of the experimental tank were covered with polyethylene sheets to minimize reflections and prevent potential visual disturbances for the fish. Both experimental tanks were equipped with two infusion systems, one on each side of the tank. The infusion systems were assigned to one of the two conditions and exchanged together with the tank according to the assigned condition. For the TNT treatment, a 100 µg/L TNT solution in holding tank water was delivered through the infusion system into the respective hideout, whereas holding tank water alone was infused for the control treatment. A total volume of 100 mL of the respective solution was placed in a separatory funnel on both sides of the experimental tank. A silicon perfusion tube connected the funnel to an injection needle attached to the inner tank wall to later deliver the solution directly into the hideout occupied by the three-spined stickleback. The flow rate of the injected solution was set to approximately 1200 mL/h using an intravenous flow regulator (Easydrop Flow Regulator - Single Scale, GVS filter technology, Bologna, Italy), so that the 100 ml infusion was completed after 5 min.

The experiment was performed double-blind to minimize bias. The experimenters applied respective treatment solutions based on labels (A/ B experiment 1; C/ D experiment 2) and were unaware whether the injected solution contained TNT or was a control. To prevent visual bias due to the yellowish color of the TNT solution, a small amount of food coloring was added to the control treatment. Treatments were alternated between individual three-spined sticklebacks to ensure balanced exposure conditions.

The experimental tank was placed within a wooden frame (60 cm × 35 cm × 60 cm). To prevent visual cues, all wooden parts were covered with black tape and a black cloth was draped over the enclosure after the three-spined stickleback was introduced into the tank. A webcam (JPL Vision Mini+, JPL Telecom, England) was centrally attached to the top of the wooden structure to enable continuous video recording of the tank throughout the experiments. Video data were recorded and stored for post hoc analysis using the Open Broadcast Studio (version 31.1.1, (OBS Project, 2024). A LED strip (neutral white, 24 V, 10 W, IP66, ~ 1000 lumens, ISOLED NEON940, ISOLED FIAI Handels GmbH, Switzerland) was installed around the outside perimeter of the tank below the water surface to achieve even illumination and to avoid reflections from the water surface to ensure clear video documentation of the behavioral response.

### Experimental procedure

For each individual exposure trial, the respective treatment-specific tank was filled with 10 L of holding tank water. Infusion solution (100 mL) containing holding tank water alone (control) or 100 µg/L TNT was prepared freshly. For each trial, a stickleback was randomly taken from a pre-assigned husbandry aquarium and placed in a white, opaque cup containing holding tank water. The stickleback was then transferred in the cup to the experimental setup.

After a settling period of five minutes in the cup, the video recording of the behavioral set up was initiated and the three-spined stickleback was transferred into the experimental tank by gently immersing the cup into the water and gradually withdrawing it to allow voluntary exit. The experimental trial started as soon as the three-spined stickleback stayed in one of the hideout zones for one minute continuously. If a fish moved to the other hideout before the one-minute waiting period had elapsed, the waiting period was restarted. Most fish completed the one-minute waiting period in their first occupied hideout, whereas a few individuals moved once or maximum twice between hideouts before the experimental trial commenced. All experimental fish successfully completed the one-minute waiting period in one of the hideouts and therefore no fish needed to be excluded from the experiment. For later analysis, the hideout zone occupied by the three-spined stickleback at the start of the observation was designated as hideout 1 zone, while the hideout located on the opposite side of the tank was designated as hideout 2 zone. Subsequently, the infusion of TNT solution or control water was initiated directly into hideout 1, marking the start of the five-minute observation period.

The observation period of five minutes was chosen based on trial experiments, as this duration allowed complete discharge of the 100 mL treatment solution into the experimental tank. Moreover, the relatively short observation time of five minutes ensured a gradual increase of TNT within the first preferred hideout (hideout 1), while an uncontaminated hideout was available throughout the observation period at the other end of the tank. The three-spined stickleback was removed from the tank and anesthetized with clove oil (150 µL of 1:10 clove oil/70% ethanol in 100 ml water; Carl ROTH). Following anesthesia, the three-spined stickleback was weighed and measured for length before being euthanized via cervical severance and gonads were examined to determine the sex of the fish.

Following each trial, the experimental tanks were rinsed thoroughly three times with clean tap water to remove residual TNT.

### Video analysis

**Fig. 2:**
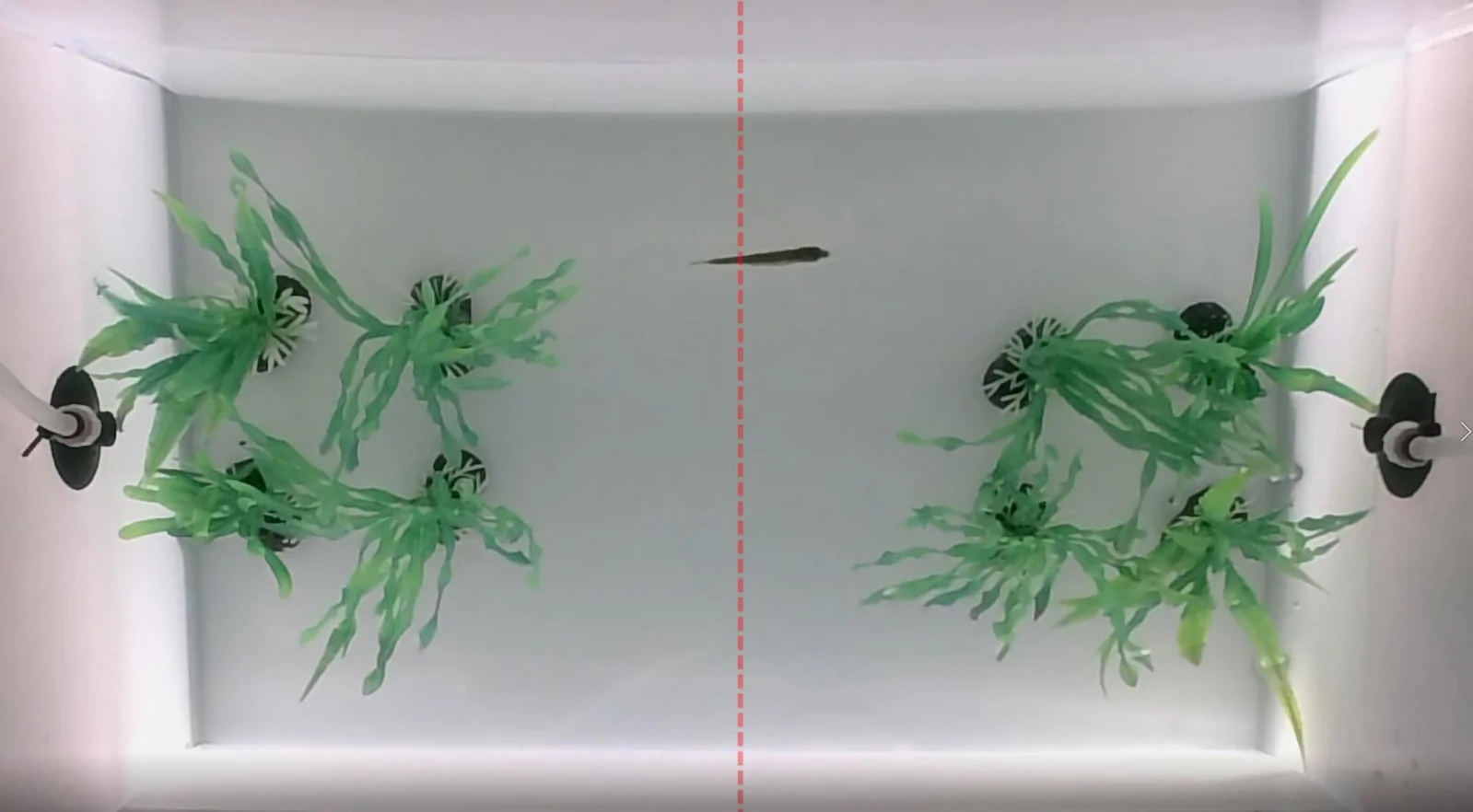
Example video analysis frame used to assess the behavioral response of sticklebacks to TNT exposure: experimental tank with a digitally added midline separating two hideout zones to count the number of line crossings. One crossing was defined as the three-spined stickleback crossing the line by more than half of its body length

The behavior of the three-spined sticklebacks exposed to either TNT solution or control water was assessed by post hoc video analysis over a five-minute observation period. For the analysis, the experimental tank was divided into two zones, each zone containing its respective hideout. When a three-spined stickleback exhibited swimming activity, movements were almost exclusively limited to direct transitions between the two hideouts rather than extended swimming across the tank. Therefore, time spent in the respective tank half in combination with crossings between tank halves best represented the swimming behavior of the tested sticklebacks. The midpoint of the tank was defined by measuring the distance between the two injection needles. As reference for the evaluation of crossings, a red line separating the two hideout zones was added for the video analysis with Shotcut (version win64-250125) (Meltytech 2024) (Fig. 2). A line crossing was counted if the three-spined stickleback crossed the red line by more than half of its body length. Furthermore, the time until the three-spined stickleback first crossed the line and left the hideout 1 zone for the first time was recorded. The total number of line crossings during the observation period was also counted as a reference for swimming activity. Moreover, the total time the three-spined stickleback spent in the hideout 1 zone was calculated by summing the time intervals that the fish was present in hideout 1 zone.

### Analysis of TNT exposure and distribution in the test setup

To assess TNT exposure of three-spined sticklebacks during the behavioral assay, TNT infusion experiments were performed without fish. In a first test, TNT distribution and potential losses during the behavioral assay were assessed using a 38-fold higher TNT concentration to ensure reliable detection. To quantify potential TNT losses within the infusion system, water samples were collected from the funnel and at the outflow. After start of infusion, additional samples were taken during the infusion at sampling points in the hideout 1 zone, the middle of the tank, and in the hideout 2 zone (supplementary Fig. A1, Table A2).

In a second test, TNT was infused at the same concentration used in the behavioral assay and water samples were taken at three sampling points across the experimental tank at 1, 2, 3, and 4 min after infusion start (Fig. A1, Table A1).

Water samples were processed and analyzed for TNT and its main metabolites (2-ADNT, 4-ADNT, 1,3,5-TNB) as described previously by Kammann et al. (2024; 2025), excluding sample preparation. Briefly, 250 µl of each water sample were mixed with 5 µl of the isotope-labeled internal standard ^13^C^15^N-TNT (2,4,6-Trinitrotoluene; 15N3,98%;13C7,99%; concentration of 0.4 ng/µL) were transferred into an insert within a 1.5 ml amber glass. Samples were analyzed by HPLC-MS/MS (Agilent 1290 Infinity High-Performance-Liquid-Chromatograph coupled to an AB Sciex QTrap 5500 Triple Quadrupole/Ion-Trap Mass Spectrometer) using an Acclaim Explosives E2 column (2.2 μm, 2.1 mm x 150 mm, Thermo Fischer Scientific). Peak detection and quantification were performed using the MultiQuant Software Version 3.0.2 (Sciex). Limits of detection (LOD, LOD_TNT_ = 0.0052 ng/mL) and limits of quantification (LOQ, LOQ_TNT_ = 0.0155 ng/mL) were calculated according to standards of DIN (Huber 1994) for TNT.

### Statistics

Statistical analysis was performed using RStudio (version 2024.12.0) to test for effects of treatment, experiment 1 and 2, sex, and their interactions on the response variables: total time (s) spent in hideout 1 zone, time (s) until hideout 1 zone was left for the first time, and number of crossings between the two hideout zones. A generalized linear model (GLM) was applied to account for the non-normal distribution of the response variables. Given the positive and right-skewed nature of the data, a Gamma distribution was selected as the appropriate error structure. The model utilized a log link function to ensure the fitted values remained positive while allowing for a multiplicative relationship between the predictor and the response variables. Model selection was automated using the dredge function, which systematically explored combinations of predictors and interactions terms to identify the best-fitting model. For total time in hideout 1 zone and time until hideout 1 zone was first left, the predictors included sex, the two experiments, treatment, and the interaction of experiment and treatment. Experiment, sex, treatment and the interaction between treatment and sex were selected as predictors for the number of crossings. Significance was assumed with p-values < 0.05.

To evaluate whether inactive individuals influenced the results, the statistical analyses were repeated after excluding sticklebacks that remained in hideout 1 throughout the entire observation period. The response variables showed positive right-skewed distributions; therefore, generalized linear models (GLMs) with a gamma distribution and log-link function were applied. The best-fitting models included experiments, sex, treatment, and the three-way interaction between these factors as predictors. As for the analyses including all individuals, the effects of experiment, sex, and TNT treatment were assessed for total time spent in hideout 1 zone, the latency to first leave hideout 1 zone, and the number of crossings between hideout zones.

## Results

### Behavior of three-spined sticklebacks during TNT infusion

The control condition included 31 females and 29 males, while 34 females and 26 males were exposed to TNT. Sticklebacks’ behavior was quantified by measuring the total time spent in the exposed hideout zone (hideout 1 zone), the latency to first transition between hideout 1 zone, and the number of crossings between the two hideout zones.

**Fig. 3:**
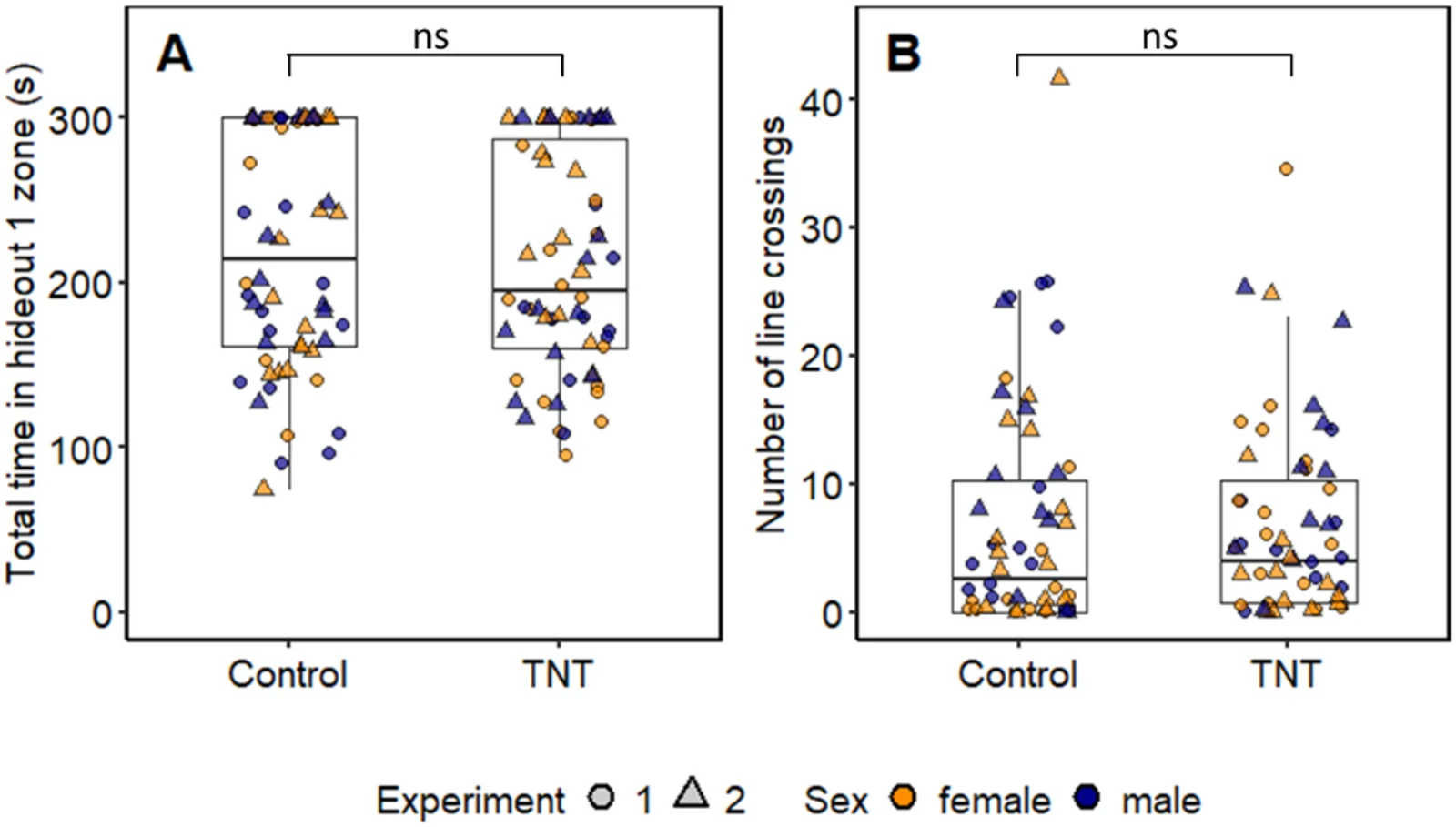
Behavioral responses of three-spined sticklebacks to TNT. (**A**) Total time spent in the hideout 1 zone. (**B**) Number of crossings between hideout zones. Each point represents an individual fish (shape = experiments 1 and 2: square = 1, triangle = 2; color = sex: orange = female, blue = male). Boxplots show medians and standard errors, ns: not significant (*p* > 0.05). Sample sizes: *n* = 60 for TNT, *n* = 60 for control. Line crossings were counted during video analysis when fish crossed the digitally added tank midline by more than half their body length. Time at the exposure site was summed over the 5-minute observation period

For all three metrics, no significant difference between the control and TNT group was detected. However, there was a non-significant trend (*p* = 0.077) that three-spined sticklebacks exposed to TNT spend less time in the exposed hideout zone compared to the control group (Fig. 3A). Notably, a fraction of the three-spined stickleback individuals remained in the infused hideout zone for the entire observation period (300 s), independent of the treatment. Further population analysis showed that in the control group, 39% of the females (12/31) and 28% of the males (8/29) remained in the infused hideout, whereas in the TNT group, this applied to 24% of the females (8/34) and 27% of the males (7/26). No significant differences (*p* = 0.192) in the time spent in hideout 1 zone were observed between male and female individuals (Fig. 3A).

Since the fraction of sticklebacks that did not leave the hideout at all might have skewed the data, data were re-analyzed with these fish excluded (supplementary Figure A2). However, statistical results with only active fish revealed no significant effects of TNT exposure on the total time in hideout 1 zone (*p* = 0.111).

To evaluate whether TNT exposure influenced the stickleback’s swimming activity, the number of crossings between both hideout zones for each individual were used as a proxy. No significant effects were found for treatment (*p* = 0.454), experiments 1 and 2 (*p* = 0.530), or sex (*p* = 0.153) on the number of line crossings (Fig. 3B). TNT treatment (*p* = 0.296), sex (*p* = 0.172), or experiments 1 and 2 (*p* = 0.243) were also not significant if inactive sticklebacks were excluded from analysis.

**Fig. 4:**
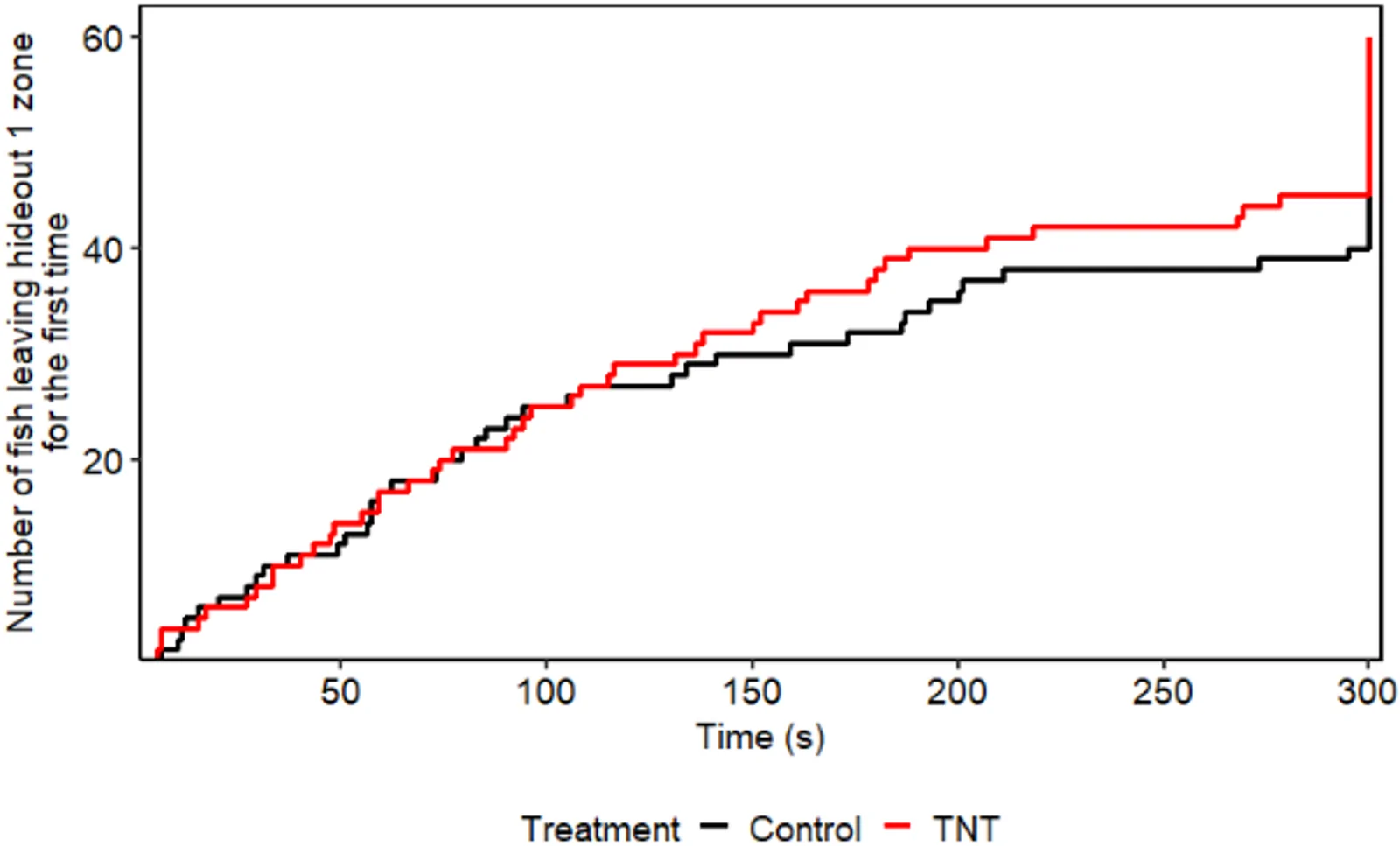
Latency to first exit from exposure site in three-spined sticklebacks under control and TNT treatment. Fish behavior was recorded for 300 s and the time until the first transition between hideout zones was determined via post-hoc video analysis. Hideout 1 zone was defined as the tank half occupied by the fish at the start of the observation period and which was infused with the respective treatment solution. Each line represents one treatment (black = Control, red = TNT). Sample sizes were *n* = 60 for TNT, *n* = 60 for control

Moreover, a potential effect of treatment, experiments or sexual differences on the sticklebacks ‘latency to first exit from the exposure site was analyzed (Fig. 4). There were no significant differences between the two experiments (p = 0.488) or between the sexes (p = 0.262). Different treatments did not significantly affect the time until the sticklebacks first crossed the line and left hideout 1 zone (p = 0.123), also if inactive sticklebacks were excluded (p = 0.180). After the initial three-minute period, slightly more stickleback exposed to TNT left the hideout 1 zone compared to the control group (Fig. 4), but this difference was not significant (p = 0.123).

### TNT distribution and recovery in experimental tank

To assess TNT recovery and distribution within the experimental tank, infusion tests were performed without fish. Recovery rate of TNT after passage through the infusion system was 90% and 68% in the entire aquarium after mixing. Part of the TNT loss was attributed to TNT metabolization into ADNTs and TNB as detected in the water (supplementary Table A3). Potential other TNT metabolites, such as DANTs, were not assessed. A further possibility for TNT loss was adsorption of TNT and its metabolites to glass or plastic surfaces.

Distribution analysis showed that TNT remained below the LOD at all sampling points during the first minute of infusion (supplementary Table A1, A2). From the second minute onward, TNT was consistently detected in the hideout 1 zone, with increasing concentrations over time. This pattern was consistent with measurements obtained using the concentration applied in the behavioral assay, with TNT concentrations in the hideout 1 zone reaching up to 1.4 µg/L (Table A1). In contrast, only low and transient detection occurred in the middle of the tank, while at the hideout 2 zone, TNT remained largely below LOD (Table A2).

## Discussion

Marine fish are facing a potential threat from corroding marine dumped munitions and are exposed to toxic munition compounds, such as 2,4,6-trinitrotoluene (TNT). Given the toxic properties of TNT, we hypothesized that fish avoid TNT at environmentally relevant concentrations to reduce exposure and associated adverse effects. In the present study, three-spined sticklebacks were exposed to gradually increasing TNT concentrations and their behavior was examined. Distinct behavioral responses of three-spined sticklebacks to the infusion with an environmentally relevant concentration of TNT (100 µg/L) were not observed. In comparison, no significant behavioral differences between three-spined sticklebacks in control and TNT treatment occurred. The present study does not provide evidence for distinct behavioral responses of fish to environmentally relevant concentrations of TNT in the water.

The present study aimed to investigate whether three-spined sticklebacks show a behavioral response to environmentally relevant TNT concentrations before exposure reaches levels associated with acute toxic effects. This question is particularly relevant in the context of marine submerged munitions, where leaking munition compounds create concentration gradients in the surrounding water (Beck et al. 2019). Beck et al. (2019) reported TNT concentrations of up to 3100 µg/L in the immediate vicinity (< 1 cm) of freely accessible gun wool material (explosive matrix). Such concentrations exceed reported lethal thresholds for fish (e.g., LD_50_: 2600 µg/L for fathead minnows) (Talmage et al. 1999; Yoo et al. 2006). However, TNT concentrations decline rapidly with increasing distance from the dumped munitions, reaching approximately 16 µg/L at 1 cm and 3.3 µg/L at 50 cm, with values depending on the sampling location and hydrodynamic conditions (Beck et al. 2019). Harmful genotoxic effects were reported in zebrafish at TNT concentrations of 100 µg/L (Koske et al. 2019). Consequently, fish approaching a munition dumpsite will encounter low TNT concentrations before reaching higher concentrations associated with acute toxic effects. Behavioral responses and potential avoidance at small doses of TNT, i.e., 1–100 µg/L, as used in the present study, could therefore reduce exposure to harmful TNT concentrations and decrease the risk of adverse effects for fish.

Higher TNT concentrations than 100 µg/L were intentionally not included in the present study, due to potential toxic effects, rising ethical concerns. Previous studies illustrated that TNT starts to become acutely toxic to fish at concentrations above 100 µg/L (Gabel and Scharsack 2026; Koske et al. 2019; Liu et al. 1983; Mariussen et al. 2018; Smock et al. 1976; Yoo et al. 2006). For the present study, the infusion concentration of 100 µg/L TNT was selected to reflect environmentally relevant conditions reported at Baltic Sea munition dump sites. Beck et al. (2019) measured TNT concentrations below 100 µg/L at decimeter distances from corroding munitions, with substantially higher concentrations only within the immediate vicinity (< 1 cm) of the munition surface. By infusing 100 µg/L, the respective concentration at decimeter distances from dumped munitions was simulated at the infusion nozzle and in hideout 1 of the present experimental setup. The testing of TNT recovery in the experimental tank during and after infusion illustrated that TNT concentrations at the hideout were initially below the limit of detection and increased gradually during exposure, reaching concentrations above 1 µg/L (Tables A1–3) and tended to accumulate near the infusion point rather than quickly diffusing across the tank. Thus, the fish were exposed to a concentration gradient comparable to that expected in the vicinity of submerged munitions. Although behavioral responses or avoidance at higher concentrations cannot be excluded, the present results indicate that three-spined sticklebacks do not exhibit a behavioral response to low environmentally relevant TNT concentrations that may be encountered before reaching highly contaminated areas near dumped munition objects.

The relatively large sample size (*n* = 120), combined with the consistent results obtained across two independent experiments, suggests that the absence of a statistically significant behavioral effect was unlikely to be attributable solely to insufficient statistical power. Although small effects may nevertheless have remained undetected, further increasing the sample size would have raised ethical concerns regarding the use of additional experimental fish, particularly given the relatively small estimated treatment effect and the consistency of the findings across experiments.

In the present study, the TNT gradient was produced by infusing TNT into the first hideout the stickleback had chosen, while a second hideout remained uncontaminated. As TNT would diffuse throughout the experimental tank over time, the observation period was intentionally kept relatively short at five minutes, to ensure that the fish had the opportunity to decide between a contaminated and an uncontaminated refuge, while maintaining its preference for shelter. Furthermore, it was expected that a behavioral response, if present, would occur relatively fast after detection of the contaminant. Finally, limiting the exposure duration reduced the risk of prolonged exposure to potential harmful TNT concentrations and was therefore consistent with ethical considerations. With the present study, we cannot rule out, that behavioral responses of fish are possible after longer exposure to low TNT concentrations. Nevertheless, it would be advantageous to the fish to respond to the contaminant as quickly as possible.

The present study cannot rule out that three-spined sticklebacks are able to detect TNT in the water, since TNT may have been detected but did not elicit a behavioral response. It is known that fish in principle can detect and response to waterborne contaminants, like heavy metals at environmentally relevant concentrations, and exhibit avoidance behavior (Araújo et al. 2016; Hartwell et al. 1987, 1989; Robinson 2009; Silva et al. 2018; Sprague 1968; Svecevičius 1999; Tierney 2016). For example, rainbow trout (*Oncorhynchus mykiss*) avoided increasing concentrations of zinc sulphate (threshold avoidance concentration 5.6 µg/L), spending more time in uncontaminated water as zinc concentrations increased (Sprague 1968). Similarly, Svecevičius (1999) showed that both rainbow trout and vimba (*Vimba vimba*) actively avoided copper- and zinc-contaminated water, whereby avoidance threshold differed between species (Svecevičius 1999). Silva et al. (2018) reported that zebrafish and guppies (*Poecilia reticulata*) exhibited avoidance behavior when exposed to copper concentrations ranging from 10 to 70 µg/L.

These previous studies employed free-choice setups similar to the present study, in which fish could freely move between a contaminated and an uncontaminated area. While fish actively avoid areas contaminated with pollutants or metals, the absence of avoidance under TNT exposure in the present study suggests that three-spined sticklebacks do not behaviorally respond to environmentally relevant TNT concentrations.

Avoidance behavior in fish was typically accompanied by increased swimming activity (Svecevičius 1999). In the present study, the number of crossings between hideout zones, as reference for swimming activity, did not show significant changes under TNT exposure. Consistent activity levels across groups did not support the hypothesis that three-spined sticklebacks respond behaviorally to environmentally relevant TNT concentrations.

The finding of the present study is consistent with observations at marine munition dump sites, where fish and other marine organisms were indeed detected near munition debris (Beck et al. 2019; Maser and Strehse 2021; Vedenin et al. 2024). Marine organisms, especially benthic organisms like starfish (*Asterias rubens)* or blue mussel, use munition shells or fragments as hard substrates, which can otherwise be scarce in deeper areas of North Sea and Baltic Sea (Edwards and Bełdowski 2016; Kampmeier et al. 2020; Vedenin et al. 2024). In addition, the piles of munitions and the wooden boxes found at the dump sites might also be attractive habitats for fish, as they provide shelter from predators, structural complexity and food availability (Della Torre et al. 2010; McDonald 2009).

In this context, the absence of avoidance behavior to TNT exposure suggests that fish inhabiting munition dump sites may experience chronic exposure to TNT. Continuous uptake of TNT by fish results in sustained exposure and prolonged risk of harmful effects. Importantly, even contamination at a concentration of 100 µg/L can have genotoxic effects (Koske et al. 2019), which could be particularly critical for the offspring of fish. If adult fish do not avoid TNT-contaminated areas during reproduction, developing embryos and larvae may be exposed to elevated genotoxic stress during sensitive life stages. Thus, fish may need to balance the ecological benefits of structured habitats at munition dump sites against the danger of sublethal, but potential long-term harmful effects.

Higher TNT concentrations may cause even more pronounced adverse effects and stronger responses in organisms, as demonstrated by Smock et al. (1976). Most of the concentrations tested by Smock et al. (1976) exceeded published LD_50_ values for fathead minnows and are known to be acutely toxic to fish (e.g.: LD_50_ 96 h at 2.7 mg/L; Yoo et al. 2006), indicating that the responses observed by Smock et al. (1976) likely reflect acute toxicity-driven shock responses rather than typical behavioral activity. Consequently, avoidance of such high concentrations could provide a protective advantage to marine organisms.

Evidence that organisms can behaviorally respond to TNT exposure to reduce potential harm has been demonstrated in other marine taxa. Schuster et al. (2021) showed that blue mussels exposed to dissolved TNT (5000–10 000 µg/L) for 96 h reduced shell opening behavior. The percentage of open shells decreased from 86% in the control group to 50% at 5000 µg/L TNT and 20% at 10,000 µg/L TNT (Schuster et al. 2021). This response suggests a protective or avoidance-related reaction to TNT exposure. However, TNT concentrations used by Schuster et al. (2021) are within a range that would presumably be acutely toxic to fish and would probably induce a shock response as described by Smock et al. (2021).

Behavioral responses in three-spined sticklebacks can be influenced by biological factors such as sex, reproductive status, and age. Male sticklebacks are generally more exploratory and bolder than females (King et al. 2013), while reproductive status also affected behavior (Kent and Bell 2018; Tinghitella et al. 2013; Ukegbu 1986). Since the reproductive status is closely associated with age, age may likewise contribute to behavioral variation (Tinghitella et al. 2013). However, these factors were unlikely to have confounded the present study. All fish were drawn from a pool of fish of the same age, reared under identical laboratory conditions, and tested as non-breeding adults. Furthermore, males and females were approximately equally represented in both the control and TNT treatment groups, and no significant sex-related differences in behavioral responses were detected. To account for differences in behavioral types, inactive fish that remained in hideout 1 throughout the entire observation period were excluded in an additional analysis to focus on more active, potentially bolder individuals. Nevertheless, no significant behavioral responses to TNT were detected. Therefore, the absence of significant behavioral responses to TNT is unlikely to be explained by differences in sex, age, reproductive status, or boldness / shyness of tested individuals.

The present study does not distinguish between the fish not detecting the contaminant or detecting it but not exhibiting a behavioral response. However, stickleback sought shelter regardless of whether the water was contaminated, suggesting that, if they detected the TNT, the benefit of shelter outweighed any potential avoidance of the contaminant. At munition dump sites in the field, habitat features such as shelter, structural complexity and food availability may attract fish despite the presence of contaminants. Individual fish that live near munition dump sites may face a trade-off situation between the potential risk of TNT exposure and the ecological benefits provided by structures at munition dump sites.

Support for such trade-offs is provided by Araùjo et al. (2016), who showed that food availability can override an avoidance response to contaminated plant effluents. Tilapia fry (*Oreochromis* sp.) avoided contaminated areas in the absence of food, whereas the presence of a food stimulus reduced the avoidance behavior and prompted movement into the contaminated areas to access higher food availability (Araújo et al. 2016). This highlights that fish may prioritize ecological benefits over avoidance of contaminants.

## Conclusion

The results of the present study demonstrated that three-spined sticklebacks do not exhibit distinct avoidance behavior or other behavioral responses to environmentally relevant concentrations of TNT. Field observations also suggest that fish do not seem to strictly avoid TNT contaminated areas near munition dump sites (Maser and Strehse 2021; Vedenin et al. 2024). In fact, TNT and its metabolites were detected in bile (Kammann et al. 2024; Maser et al. 2023, 2024) and in urine (Kammann et al. 2025) of flat fish near munition dump sites. The present study suggests that fish do not behaviorally respond to environmentally relevant TNT concentrations in water in the presence of structures providing shelter. As marine munition dump sites provide structurally complex habitats that attract fish, the ecological benefits of these structures may outweigh avoidance of TNT contamination. Consequently, fish are likely to remain exposed to munition compounds, and this exposure may even increase as corrosion progresses. Therefore, regular monitoring of munition contaminants in fish is essential to assess potential risks to fish health and seafood consumers and to identify potential long-term effects of TNT exposure. 

If fish and other marine organisms do not avoid munition dump sites, the clearance and remediation of dumped munitions represent important management strategies to reduce current and future contaminant exposure.

## Supplementary Information

Below is the link to the electronic supplementary material.

Supplementary Material 1

## Data Availability

Research data were deposited into the PANGEA data base and are available at the following: Willmer, Joy; Schubert, Hendrik; Hölzel, Maj-Britt; Stegmann, Niklas; Kammann, Ulrike K R; Scharsack, Jörn Peter (publication year): Observational dataset of behavioral response of three-spined sticklebacks to TNT exposure in a closed laboratory setup [dataset]. PANGAEA, https://doi.pangaea.de/10.1594/PANGAEA.990263.
