# Supplementary material for: Three-spined sticklebacks do not respond behaviorally to environmentally relevant concentrations of the toxic explosive 2,4,6-trinitrotoluene
Source: Ecotoxicology. 2026 Oct 23;35(9):199. doi: 10.1007/s10646-026-03169-7 (PMC13694530; doi:10.1007/s10646-026-03169-7)
Supplement: Supplementary file 1 [file 10646_2026_3169_MOESM1_ESM.docx]

Appendix to Wilmer et al., Three-spined sticklebacks do not respond behaviorally to environmentally relevant concentrations of the toxic explosive 2,4,6-trinitrotoluene

**Fig. A1**: **Experimental setup for TNT concentration measurements during infusion to assess TNT distribution.** Water samples were collected through tubes in hideout 1 zone (5 cm distance to inflow in hideout 1), in the middle of the tank (20 cm distance to inflow) and in hideout 2 zone (35 cm distance to inflow) during infusion of the TNT-solution. Distribution was assessed in two infusion tests using either the concentration applied in the behavioral assay (100 µg/L, Table A1) or a 38-fold higher concentration (3848 µg/L, Table A2) to ensure reliable detection.

**Table A1: Spatial distribution of TNT during the behavioral assay.** TNT concentrations (µg/L) were measured in hideout 1 zone, in the middle of the tank, and in hideout 2 zone (Fig. A1) during infusion with 100 mL TNT solution (100 µg/L) into 10 L tank. Limit of detection (LOD_TNT_ = 0.35 µg/L) and quantification (LOQ_TNT_ = 1.04 µg/L) were calculated according to standards of DIN (Huber, 1994) for TNT.

| **Sampling Time**  (min) | **Hideout 1 zone**  (µg/L) | **Middle of tank**  (µg/L) | **Hideout 2 zone**  (µg/L) |
| --- | --- | --- | --- |
| 1 | < LOD | < LOD | < LOD |
| 2 | 1.13 | < LOD | < LOD |
| 3 | 1.17 | < LOD | < LOD |
| 4 | 1.42 | < LOD | < LOD |

**Table A2: Spatial distribution of TNT in experimental tank.** TNT concentrations (µg/L) were measured in hideout 1 zone, in the middle of the tank, and in hideout 2 zone (Fig. A1) during and after infusion of 100 mL TNT solution (3848 µg/L = 38-fold test concentration) into 10 L tank water. After complete infusion, the tank was mixed and three additional samples were taken at each position. Limit of detection (LOD_TNT_ = 0.0052 µg/L) and quantification (LOQ_TNT_ = 0.0155 µg/L) were calculated according to standards of DIN (Huber, 1994) for TNT.

| **Sampling Time (min)** | **Hideout 1 zone** (µg/L) | **Middle of tank** (µg/L) | **Hideout 2 zone** (µg/L) |
| --- | --- | --- | --- |
| 0 | < LOD | < LOD | < LOD |
| 0.5 | < LOD | < LOD | < LOD |
| 1 | 0.74 | < LOD | < LOD |
| 2 | 0.15 | 0.15 | 0.08 |
| 3 | 30.24 | 0.06 | < LOD |
| 4 | 61.53 | 0.45 | < LOD |
| After homogenization | 21.03 | 22.46 | 21.86 |

**Table A3: Spatial distribution of TNT metabolites (µg/L) in experimental tank corresponding to amounts of TNT given in Table A2.** Water samples were collected in hideout 1 zone, in the middle of the tank, and in hideout 2 zone (Fig. A1) during and after infusion of 100 mL TNT solution (3848 µg/L = 38-fold test concentration) into 10 L tank water. Limits of detection (LOD_2-ADNT_ = 0.002 µg/L; LOD_4-ADNT_ = 0.0015 µg/L; LOD_1,3,5-TNB_ = 0.0783 µg/L) and limits of quantification (LOQ_2-ANDT_ = 0.006 µg/L; LOQ_4-ADNT_ = 0.0044 µg/L; LOQ_1,3,5-TNB_ = 0.2348 µg/L) were calculated according to standards of DIN (Huber, 1994) for TNT.

|  | 2-ADNT  (µg/L) | | | 4-ADNT  (µg/L) | | | | | 1,3,5-TNB  (µg/L) | | | | |  |
| --- | --- | --- | --- | --- | --- | --- | --- | --- | --- | --- | --- | --- | --- | --- |
| **Sampling time (min)** | **Hideout 1 zone** | **Middle of tank** | **Hideout 2 zone** | |  | **Hideout 1 zone** | **Middle of tank** | **Hideout 2 zone** | |  | **Hideout 1 zone** | **Middle of tank** | **Hideout 2 zone** | |
| 0 | 0.104 | 0.013 | 0.009 | |  | 0.119 | 0.026 | 0.024 | |  | 1.803 | 1.479 | < LOD | |
| 0.5 | 0.123 | 0.004 | 0.006 | |  | 0.306 | 0.032 | 0.004 | |  | 2.272 | 0.330 | < LOD | |
| 1 | 0.011 | 0.196 | 0.007 | |  | 0.017 | 0.265 | 0.029 | |  | 0.327 | 1.075 | 0.050 | |
| 2 | 0.011 | 0.054 | 0.012 | |  | 0.005 | 0.277 | 0.025 | |  | < LOD | 6.729 | < LOD | |
| 3 | 0.088 | 0.009 | 0.056 | |  | 0.091 | 0.015 | 0.156 | |  | 0.858 | 0.207 | < LOD | |
| 4 | 0.168 | 0.015 | 0.130 | |  | 0.246 | 0.017 | 0.225 | |  | 0.704 | < LOD | 0.278 | |
| After homogenization | 0.210 | 0.233 | 0.206 | |  | 0.098 | 0.211 | 0.232 | |  | 7.768 | 1.288 | 6.665 | |
|  |  |  |  | |  |  |  |  | |  |  |  |  | |

**Fig. A2: Behavioral responses of three-spined sticklebacks to TNT after exclusion of inactive individuals which did not leave hideout 1.** Fish that did not leave hideout 1 during the entire five-minute observation period were removed from this analysis. (A) Total time spent in the hideout 1 zone. (B) Number of crossings between hideout zones. Each point represents an individual fish (shape = experiments 1 and 2: square = 1, triangle = 2; color = sex: orange = female, blue = male). Boxplots show medians and standard errors, ns: not significant (p > 0.05). Sample sizes: n = 45 for TNT, n = 40 for control. Line crossings were counted during video analysis when fish crossed the digitally added tank midline by more than half their body length. Time at the exposure site was summed over the 5-minute observation period.
